# Harnessing the Angiogenic Potential of Stem Cell-Derived Exosomes for Vascular Regeneration

**DOI:** 10.1155/2016/3409169

**Published:** 2016-04-03

**Authors:** F. Alcayaga-Miranda, M. Varas-Godoy, M. Khoury

**Affiliations:** ^1^Laboratory of Nano-Regenerative Medicine, Faculty of Medicine, Universidad de Los Andes, 7620001 Santiago, Chile; ^2^Cells for Cells, 7620001 Santiago, Chile; ^3^Laboratory of Reproductive Biology, Faculty of Medicine, Universidad de Los Andes, 7620001 Santiago, Chile; ^4^Consorcio Regenero, Chilean Consortium for Regenerative Medicine, 7620001 Santiago, Chile

## Abstract

Mesenchymal stem cells (MSCs) are known to display important regenerative properties through the secretion of proangiogenic factors. Recent evidence pointed at the key role played by exosomes released from MSCs in this paracrine mechanism. Exosomes are key mediators of intercellular communication and contain a cargo that includes a modifiable content of microRNA (miRNA), mRNA, and proteins. Since the biogenesis of the MSCs-derived exosomes is regulated by the cross talk between MSCs and their niche, the content of the exosomes and consequently their biological function are dependent on the cell of origin and the physiologic or pathologic status of their microenvironment. Recent preclinical studies revealed that MSCs-derived exosomes have a critical implication in the angiogenic process since the use of exosomes-depleted conditioned medium impaired the MSCs angiogenesis response. In this review, we discuss the current knowledge related to the angiogenic potential of MSCs-exosomes and methods to enhance their biological activities for improved vascular regeneration. The current gain of insight in exosomes studies highlights the power of combining cell based therapies and their secreted products in therapeutic angiogenesis.

## 1. Introduction

Mesenchymal stem cells (MSCs) are self-renewing progenitor cells located within the stroma of the bone marrow (BMSCs) and other organs, including adipose tissue (AT-MSCs), postnatal tissues, such as umbilical cord (UC-MSCs) and placenta (PL-MSCs), or menstrual fluid (MenSCs) [1, 2]. MSCs possess outstanding potentials due to their reported regenerative potency. Currently, they are being clinically investigated against an increasingly wide spectrum of disease indications [3]. The cell therapy field has witnessed recently an important expansion of the uses of MSCs in clinical trials. This was manifested through a significant increase of the number of Investigational New Drug (IND) submissions to the FDA related to MSCs-based product between 2006 and 2012 [4], with around 500 trials enrolled in the ClinicalTrials.gov database (https://www.clinicaltrials.gov/, queried in January 2016).

Despite the demonstrated immunomodulatory, angiogenic, and regenerative properties of MSCs* in vitro* and* in vivo*, incomplete knowledge about their mechanism of action is still under debate. However, the assumption that MSCs therapeutic efficacy occurs after initial engraftment and subsequent differentiation into appropriate cell types has been almost dismissed since their biologic effect is independent of the proximity of the transplanted cells to the injured tissue [3, 5–7]. Currently, it has been well documented that MSCs exert their biological/therapeutical activity through a paracrine effect mediated by the release of small molecules as growth factors, cytokines, and chemokines [8]. In addition to secreting an array of soluble factors, it has also been recognized that MSCs release large numbers of extracellular vesicles (EVs), which have emerged as an important mediator of cell-to-cell communication that is involved in normal physiological process and also in the development and progression of diseases [9].

Among the many subtypes of EVs, endosome-derived exosomes have emerged as physiologically relevant and powerful components of the MSCs secretome's functionality [10]. Exosomes act as key agents in intercellular communications through transfer of information via their cargo, which includes proteins, DNAs, messenger RNAs, and microRNAs (miRNAs) [11–13]. Growing evidence has shown that the therapeutical effect of MSCs-derived exosomes in pathological conditions, as ischemic heart disease, kidney injury, wound healing, and modulating the tumor angiogenesis, is similar to the functional effect of their parent cells, indicating the active biological/therapeutical role of MSCs-derived exosomes in, at least, these diseases [8, 14–18]. In consequence, MSCs-derived exosomes are gaining momentum in the field as a putative surrogate to MSCs-based therapeutics with a stronger safety profile [19].

The role of MSCs in directing and promoting angiogenesis still needs further investigation, but presumably their exosomes are involved in this process since the use of conditioned medium depleted of exosomes impaired the angiogenesis response [14]. Previous data have proposed that EVs proteins such as sonic hedgehog [20] and platelet derived growth factor (PDGF) [21] or the transfer of mRNA and miRNAs [22] might contribute to the proangiogenic activity [23]. Furthermore, new data have identified nuclear factor *κ*B (NF*κ*B) signaling as a key mediator of MSCs-exosome-induced angiogenesis in endothelial cells [19]. In this review, we summarize and discuss the current knowledge about the biological activity of MSCs-derived exosomes in the angiogenic process, with a special emphasis on their potential therapeutical uses as agent to modulate angiogenesis.

## 2. Exosomes

In 1983, Pan and Johnstone described for the first time the EVs in maturing sheep reticulocytes [24]. Initially, the release of EVs was thought to represent a disposal mechanism by which cells eliminate unwanted proteins and other molecules [9]. Currently, it is known that EVs are key mediators of the intercellular communication which may reprogram target cells by delivering functional mRNA and miRNA sequences and proteins [25]. The best characterized and also the most numerous EVs class is the exosomes [9], which have a diameter of 40–120 nm and a flotation density of 1.13–1.19 g/mL in a sucrose gradient and have exosome-associated markers [3, 11]. Preparations of EVs, typically a mixture of exosomes and other subtypes, can be isolated from all types of body fluids as blood, urine, bronchoalveolar lavage fluid, breast milk, amniotic fluid, synovial fluid, pleural effusions, and ascites and can also be purified from culture supernatants of many cell types, including MSCs (reviewed in [9]). In Table 1, the basic characteristics of the main EVs subtypes are described: apoptotic bodies, microvesicles, and exosomes.

### 2.1. Characteristics of Exosomes

Exosomes are small membrane-bound vesicles with remarkable physiological properties [25]. They are secreted by a myriad of cells types and found in essentially all biological fluids, and they originate from inward budding of late endosomes with resultant multivesicular bodies that are fused with the plasma membrane [26]. After the fusion, exosomes are released into the extracellular milieu and can be either taken up by target cells residing in the microenvironment or carried to distant sites through biological fluids [10]. Exosomes from different cellular origins sequester a common set of molecules that are essential for their biogenesis, structure, and trafficking, as well as cell-type-specific components which, presumably, reflect the biological function of the parent cell [27]. Exosomes transport characteristic protein and lipid signatures, and they also package nucleic acids, mainly many RNA species with regulatory functions [28].

Exosomes are unique in their protein and lipid composition, providing characteristics that reflect their cellular source. They are rich in annexins, tetraspanins (CD63, CD81, and CD9), and heat-shock proteins (such as Hsp60, Hsp70, and Hsp90), expose low amounts of phosphatidylserine, and include cell-type-specific proteins [29, 30]; for example, CD80 and CD86 are expressed on dendritic cell-derived exosomes [31], and CD19 is expressed on B-cell-derived exosomes [32].

Since the exosomes function is dependent on their internalization in the target cells, different types of interaction have been hypothesized: exosomes may associate with the plasma membrane via ligand-receptor interactions [33] or lipids as phosphatidylserine [34]. The internalization can occur through direct fusion of the exosomes with the plasma membrane, leading to the release of the exosomal cargo into the cytoplasm of the cell. Instead, they also can enter into the target cells by receptor-mediated endocytosis and later fuse with the limiting membrane of the endosome releasing the exosomal content to be recycled to the cell surface or to be degraded in the lysosome [33, 35, 36].

As mentioned previously, exosomes enclose a wide variety of molecules which are recorded in the ExoCarta database (http://www.exocarta.org/) [37], with a similar signature of their parent cells. Although the molecular compositions of exosomes are cell-dependent, the incorporation of some molecules (especially miRNA) into the exosomes is not random and it could be modified under different physiological or pathological conditions, which suggest that changes in the microenvironment induce a modification of the cell content that is consequently transmitted to the exosome cargo. The miRNA analysis performed by Zhang and colleagues [38] in several cell lines revealed that a subset of miRNAs, as miR-150, miR-142-3p, and miR-451, enter preferentially the exosomes [39]. In addition, the comparison of the expression levels of let-7 miRNA family members in gastric cancer cell line-derived exosomes with respect to lung, colorectal, and stomach cancer cell line-derived exosomes showed that let-7 miRNA family are abundant in gastric cancer-derived exosomes but are less abundant in exosomes derived from other cancer cells [40]. Also, in pathological conditions, exosomes possess a different miRNA signature. It has been reported that the level of miRNA-21 in serum EVs in glioblastoma patients increases in comparison with healthy donors [41]. In contrast, levels of let-7f, miR-20b, and miR-30e-3p are lower in vesicles from the plasma of non-small-cell lung carcinoma patients than normal controls [42].

### 2.2. Specific Characteristics of MSCs-Derived Exosomes

Similar to other cell types, MSCs secrete exosomes with outstanding properties, enclosing their peculiar signature. As was described by Lai and colleagues [3], exosomes are known to function primarily as “one-way agents” of cellular components from the secreting cells to the target cells to modulate the latter's activities. Presumably, MSCs-derived exosomes function in a similar way, specifically as communication agents released by MSCs to effect the stromal support functions of MSCs through the maintenance of a dynamic and homeostatic tissue microenvironment. Likewise, MSCs secrete EVs differently depending on external stimulation, which suggests that the biogenesis of EVs is a process regulated by the cross talk between MSCs and their microenvironment [9, 43, 44]. Thus, hypoxia or inflammatory conditioning of MSCs has been shown to regulate protein packaging into EVs, affecting in this way their functional properties [9, 43, 44]. MSCs-derived exosomes have the same morphological features and common surface markers with respect to exosomes secreted by other cells. However, they contain critical adhesion molecules, as CD29, CD44, and CD73, and signaling molecules characteristic of the MSCs [21, 29].

An interesting study performed by Baglio and colleagues [10] to define the exosome-enclosed RNA species of two different sources of MSCs revealed that AT-MSCs and BMSCs have highly similar small RNA expression profiles dominated mainly by miRNAs and small nucleolar RNAs, of which 150–200 miRNAs are present at physiological levels. In contrast, the miRNA pool in MSCs-exosomes represents only ~2–5% of the total small RNAome. Importantly, the authors also determined that the miRNAs in exosomes do not conservatively reflect the cellular content as a defined set of miRNAs are overrepresented in exosomes (miR-4485; miR-150-80; miR-486-5p; and miR-6087) compared to the parent cell. Also, differences between AT-MSCs and BMSCs EVs (miR-486-5p, miR-10a-5p, miR-10b-5p, miR-191-5p, and miR-222-3p in AT-MSC exosomes and miR-143-3p, miR-10b-5p, miR-486-5p, miR-22-3p, and miR-21-5p in BMSC exosomes) suggest that the tissue-specific microenvironment might influence the exosomal sorting of the MSCs raising the possibility that AT-MSCs and BMSCs might deliver different information into their microenvironments and, consequently, exert different effect in their target cells. In fact, previous data of Del Fattore and colleagues [45] demonstrated differential effects of EVs secreted by BMSCs, UC-MSCs, and AT-MSCs on glioblastoma U87 MG cells. Although no molecule responsible for these controversial effects was identified in the study, the authors demonstrated that BM- and UC-MSC-EVs decreased cancer cell proliferation, while an opposite effect was observed with AT-MSC-EVs. Moreover, both BM- and UC-MSC-EVs induced apoptosis of glioblastoma cells, while AT-MSC-EVs had no effect.

## 3. MSCs-Derived Exosomes and Angiogenesis

Angiogenesis is the formation of new blood vessels from the preexisting vasculature. Blood vessel formation is fundamental to development, while its dysregulation can contribute to serious disease [52]. One of the most interesting debates regarding MSCs concerns their angiogenic potential [53], which can vary depending on the source of origin [1, 2, 54]. The reason for this is unknown, but the stem cell niche of origin represents a determinant factor to be considered since the classic MSCs properties, such as their immunomodulatory, differentiation, and paracrine activities, can be highly affected by changes in their microenvironment [55].

As was reviewed by Watt and colleagues [52], although the relevance of the MSCs differentiation into endothelial cells is still under debate, it is commonly accepted that transplanted MSCs promote angiogenesis mainly by secreting paracrine or trophic factors. Although the complete MSCs secretome is not fully characterized, factors released by MSCs possess an important angiogenic activity [23]; exosomes have been proposed as key agents in the modulation of the angiogenesis since the use of conditioned medium depleted of exosomes impaired the angiogenesis response [14]. In addition, MSCs-derived exosomes are uptaken by human umbilical vein endothelial cells (HUVECs), resulting in a dose-dependent enhancement of* in vitro* proliferation, migration, and tube formation of endothelial cells, which might be one of the critical processes in the new vessel formation [14, 49].

In a recent report, Anderson and colleagues [19] demonstrated that either canonical secretory proteins or exosomally delivered proteins are drivers of the MSCs secretome's functionality, which in turn is influenced by the microenvironmental changes. In the study, the exposure of MSCs to a peripheral arterial disease- (PAD-) like microenvironment increases the expression of several proangiogenic signaling associated proteins including epithelial growth factor (EGF), fibroblast growth factor (FGF), and PDGF. Furthermore, the exposure of MSCs to a PAD-like microenvironment induces elevated exosome secretion, which contain a robust angiogenic signaling profile and are capable of inducing angiogenesis* in vitro* via the nuclear factor kappa-light-chain enhancer of activated B-cells (NF*κ*B) pathway [19].

Also, it has been reported that PDGF regulates the secretion of EVs by AT-MSCs, changes their protein composition, and enhances their angiogenic potential [56]. PDGF stimulated the secretion of AT-MSC-EVs with* de novo* expression of proangiogenic molecules as c-Kit and its ligand stem cell factor (SCF) and with the absence of antiangiogenic molecules such as angiostatin and endostatin [56]. Since the c-Kit, a tyrosine kinase receptor expressed by progenitor cells, plays a key role in the amplification and mobilization of progenitor cells, EVs carrying c-Kit might recruit endothelial progenitor cells at the site of tissue remodeling [56]. Likewise, SCF/c-Kit signaling promotes the survival, migration, and capillary tube formation of HUVECs [57] and recruitment of MSCs [58]. The observation that blockade of c-Kit and SCF significantly reduced the angiogenic potential of PDGF-EVs suggested a contribution of these factors to EV-induced angiogenesis [56].

miRNAs are small noncoding RNA molecules known to regulate several processes including angiogenesis [13]. miRNAs have been implicated as important exosomal components and largely determine the effects of exosomes on target cells [59]. The release of these miRNAs by MSCs could play a role in the stem cell niche maintenance by controlling and fine-tuning the proliferation, differentiation, and homing of cells [10]. In fact, several miRNAs highly represented in MSCs-exosomes modulate angiogenesis (miRNA-222, miRNA-21, and let-7f) and endothelial cell differentiation (miRNA-6087) [10]. In consequence, the internalization of these miRNAs at sites of injury can stimulate the proliferation of cells and promote the angiogenesis for tissue repair [10].

## 4. Enhancing the Angiogenic Potential of Exosomes

One approach to enhance the angiogenic activity of exosome-based therapies is to screen for the highest cargo content of proangiogenic factors among different cell types and sources. As an example, exosomes derived directly from vessel related cells such as the endothelial progenitor cells (EPC) possess a high angiogenic potential that can be explained by their natural physiological function. Indeed, EPC-derived exosomes accelerated the reendothelialization after endothelial damage in the rat carotid artery and endothelial cells stimulated with these exosomes showed increased expression of proangiogenic factors [60]. We have previously published that haploidentical MSCs from different placental tissues possess different properties [2], suggesting that the biological activity of MSCs-derived exosomes is variable depending on the tissue of origin. The selection of the optimal proangiogenic source can be performed through screening of the different MSCs origins in tubule formation and plug transplantation assays* in vitro* and* in vivo*, respectively [1]. In addition to the screening strategy mentioned above, there are many cell conditioning and modification procedures that are currently being investigated in order to further enhance the angiogenic activity of exosomes.

### 4.1. Stress-Induced Enhancement

Exosomes are not the cell's “mini-me”: the molecular composition of exosomes is not an identical representation of the cell at a smaller scale. On the contrary, exosomes are enriched with specific RNAs or proteins, while other molecules are absent, indicating the existence of a specialized mechanism controlling the loading of molecules into exosomes. The exosome composition does not remain unchanged and can be altered under different biological and microenvironmental changes affecting the cell. This suggests that the incorporation of cargo is a regulated but also a modifiable process. However, the mechanisms that control the fluctuations in the exosome cargo affected by the cellular state changes are still elusive. Application of stress situation such as hypoxia, irradiation, or oxidative stress can alter the exosomes content and thus their physiological function.

Stress situations are frequent in organ injuries and need to be overcome to enable efficient tissue regeneration. Exosome-mediated signaling is thus affected by a variety of stress conditions. The stress-induced changes in exosomal RNA and protein repertoire are thought to provide protective bystander signals for target injured cells. In fact, Borges and colleagues showed that hypoxic injury/stress causes injured epithelial cells to increase the production of exosomes and to also alter their composition to facilitate angiogenesis and tissue repair through a TGF-beta mediated mechanism [61]. Likewise, exosomes derived from brain tumor glioblastoma cells grown at hypoxic compared with normoxic conditions induce angiogenesis by potentiating the endothelial cells to secrete an enhanced level of growth factors and cytokines [62].

**Figure 1:**
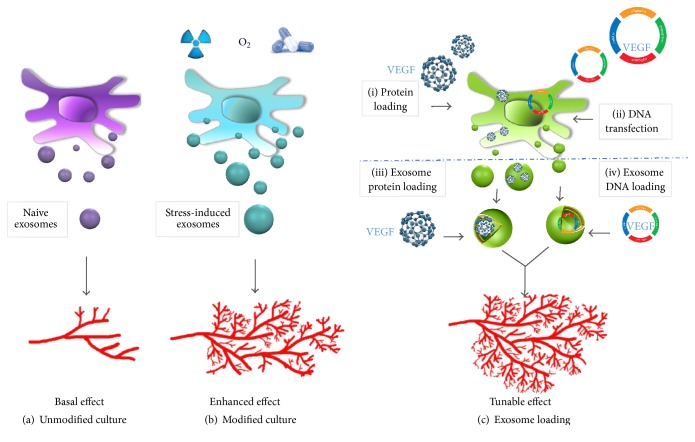
Different possible strategies to enhance the angiogenic potential of exosomes released by stem cells. (a) Stem cells cultured under normal culture condition constitutively produce exosomes with a basal angiogenic potential. (b) Specific* in vitro* stress conditions mimicking organ injury situations, such as hypoxia, irradiation, or drug treatments, induce changes in exosomal RNA and protein repertoire. These alterations of exosomal composition are thought to facilitate angiogenesis and tissue repair through an enhanced level of growth factors and cytokines. (c) The transfection of exosomes producer cells with protein or DNA encoding therapeutically active angiogenic compounds which are then released within the released exosomes constitutes another approach. Since overexpression of a specific factor does not ensure a similar increase of its representation in the exosome cargo, another strategy is to directly load the exosomes after release with proangiogenic factors such as the recombinant VEGF protein or the vector encoding its expression. Hence, boosting the exosome with proangiogenic factors can be achieved directly or indirectly, and each approach has its advantages and limitations and may be dictated by the type of the encapsulated molecule (molecular weight, posttranslational modifications) and conditions suitable for a specific type of exosome-encapsulated cargo.

Following the successful infusion of young GVHD patients with exosomes with no detectable side effects [63], the clinical applications of exosomes are increasingly anticipated. While manufacture upscaling strategies and clinical grade batches are required, culture conditions can also be modified to enhance the basal angiogenic effect of exosomes (Figure 1). It has been shown that MSCs-derived microvesicles released upon hypoxia stimulation were promptly uptaken by HUVECs and were able to promote angiogenesis in a myocardial infarction model [49].

Besides hypoxic conditions, it was found that exosomes isolated from irradiated conditioned medium could also induce bystander effects. There was a 78% increase of the protein cargo identified in exosomes from irradiated cells in comparison to control cells. The proteins specifically overrepresented in the radiation-induced exosomal cargo were those involved in transient suppression of transcription and translation or stress-induced signaling. The specific effect of irradiation on different angiogenic factors remains to be well characterized. Other stress conditions that might present an interest for enhancing the angiogenic potential of exosomes include PH variation, calorie restriction, and drug pretreatments [64]. While tuning the culture conditions represents an indirect process to modulate the exosomes' cargo and therefore enhances their angiogenic activity, this process is considered an uncontrollable method. In fact, while culture condition modification can enhance the expression of the factors of interest (proangiogenic), it can also influence a large panel of proteins involved in various pathways. This can result in creating number of uncontainable side-effects. Henceforth, exogenous protein or drug loading strategies are currently being developed to harness the exosome potential in a more tunable approach.

### 4.2. Loading of Angiogenic Factors

Presently, several distinct methods can be exploited for the loading of therapeutic cargo; in this particular application, the goal would be to tune their angiogenic potential. A first approach consists of loading donor cells with a proangiogenic drug, which is then released in exosomes. Many proofs of concept were performed in this regard by charging exosome with different types of drugs or nanovesicles [65]. Various passive and active methods have been tested for the drug loading of exosomes including electroporation, saponin, extrusion, and dialysis.

In a model where porphyrins were employed as model drugs, exosome-loaded drugs showed a significant increase of the cellular uptake when compared to free or liposome encapsulated drug. Furthermore, exosome loaded with hydrophilic porphyrins induced a stronger phototoxic effect than free drug in a cancer cell model [66]. Using a similar approach, the angiogenic potential can be enhanced by adding factors such as VEGF to their cargo. The control of the loaded quantities will enable the tuning of the level of the desired effect.

As shown in Figure 1, the transfection of exosomes producer cells with protein or DNA encoding therapeutically active angiogenic compounds which are then released in exosomes constitutes another approach. However, overexpression of a specific factor does not ensure a similar increase of its representation in the exosome cargo, as it is dependent on different mechanism involved in the protein packaging mechanism of exosomes as described previously.

Boosting the exosome with proangiogenic factors can be achieved directly or indirectly, and each approach has its advantages and limitations and may be dictated by the type of the encapsulated molecule (molecular weight, posttranslational modifications) and conditions suitable for a specific type of exosome-encapsulated cargo. Finally, different head-to-head comparisons are necessary to define the most efficient loading strategy.

## 5. MSCs-Derived Exosomes and Their Therapeutic Effects

From a translational clinical point of view, exosomes secreted from MSCs have shown encouraging therapeutic effect in various preclinical models [8, 16–18, 23, 56], indicating that effectively exosomes transport key therapeutical molecules that might stimulate the proliferation of different cells and induce angiogenesis for tissue repair. In addition, exosomes isolation is potentially sustainable and reproducible [9] and their use instead of stem cells could represent a therapeutic strategy due to the presence of important advantages as higher stability, no risk of aneuploidy, lower possibility of immune rejection, and resistance to the influence of the microenvironment [29]. In Table 2, we summarize the prior data of* in vivo* works on MSCs-derived exosomes in different pathologic conditions.

### 5.1. MSCs-Exosomes and Cardiovascular Diseases

Cardiovascular diseases (CVD) are the main cause of death in the world [67] and many efforts to find the best therapy to improve the outcome of these diseases have been done. In the last decade, stem cells based therapy has shown important advance in the field [68]. Among the different types of stem cells, MSCs and their paracrine factors are considered as a potential treatment for CVD [69–71]. Studies performed in murine and porcine preclinical model of myocardial infarct showed that MSCs-derived conditioned medium reduces the infarct size [72]. It was later on evidenced that the main component accounting for the observed biological effect was the exosomes contained in the conditioned medium [8]. To date, evidence shows the therapeutic benefit of exosomes derived from MSCs in CVD, and one of the reasons of their effect is attributed to the proangiogenic properties present in exosomes.

Bian and collaborators found that EVs isolated from BMSCs under hypoxic conditions promote blood vessel formation to protect the cardiac tissue from ischemic injury [49]. Furthermore, Teng and colleagues confirmed these results by showing that BMSCs-derived exosomes stimulate the neovascularization with an improvement in the cardiac function in a rat myocardial infarction model [50]. In a separate work, exosomes derived from UC-MSCs also showed protective effects on acute myocardial infarction promoting angiogenesis [51]. Also, MSCs from different origin showed a similar potential. In fact, exosomes isolated from AT-MSCs induced* in vitro* vessel-like structure formation and* in vivo* vessel formation in human microvascular endothelial cells [56]. In the same scope, PL-MSCs-exosomes promoted migration and angiogenic tube formation of endothelial cell contributing to the vascular adaptation due to the hypoxic condition [43]. These lines of evidence support the idea that exosomes derived MSCs, regardless of their origin, could be used as a treatment for CVD. To strengthen insights into how exosomes might work in patients, proteomic analysis of exosomes from MSCs cultured under ischemic conditions was performed, and the possible effectors involved in the induction of angiogenesis were identified. The list included PDGF, EGF, and FGF, where the modulation of angiogenesis was proposed to be mediated by the NF*κ*B pathway [19].

### 5.2. MSCs-Derived Exosomes and Tumor Angiogenesis

Angiogenesis is an essential step for the growth of cancer. Tumor cells frequently overexpress proangiogenic factors, such as VEGF, for their progression. Since MSCs exhibit enhanced natural tropism for tumors, several studies have investigated the effect of MSCs in the tumor angiogenic microenvironment. These studies have reported controversial angiogenic properties: while some reports showed that MSCs support tumor angiogenesis [73–75], other studies observed a range of antiangiogenic properties [76–78]. In line with these observations, exosomes derived from different MSCs origins have also shown paradoxical angiogenic properties. It has been reported that MSCs-secreted exosomes exert both pro- and antiangiogenic effects mediating the up- and downregulation of* VEGF* expression in cancer cells, respectively [15, 17]. Although these discrepancies are not completely understood, they can be explained by the variability of the responses depending on the different tumor types. Also, the heterogeneity of MSCs, which determine the exosomal cargo of MSCs-derived exosomes, plays an important role in their angiogenic effect. Consistent with these observations, unpublished data from our laboratory also demonstrate an opposite effect of exosomes secreted by BMSCs and MenSCs on tumor angiogenesis. Our results showed that MenSCs-derived exosomes possess an antitumoral activity in cancer cells by blocking tumor-induced angiogenesis in a ROS-dependent mechanism. This activity is specific to the menstrual cells source as opposite effects were observed with BMSCs-exosomes (Alcayaga-Miranda and colleagues, manuscript under revision).

Lee and colleagues [15] reported that murine (m) BMSCs-secreted exosomes significantly downregulated the mRNA and protein level of VEGF in a mouse breast cancer cell line (4T1) in a concentration-dependent manner, resulting in the inhibition of the proliferation and migration of endothelial cells and, consequently, the suppression of the angiogenesis. Also, the study showed that these exosomes were enriched with miRNA-16, known to target VEGF. Hence, the transfer of miRNA-16 into the tumor cells resulted in a decreased expression of VEGF in 4T1 cells.

In contrast, another study showed that exosomes released by human BMSCs promote the increase of VEGF in gastric carcinoma SGC-7901 cells also in a dose-dependent manner, through activation of ERK1/2 and p38 MAPK pathways, which regulate the VEGF expression. In this study, the authors showed that blocking ERK1/2 activation reversed the increased expression of VEGF levels induced by BMSC-exosomes. Moreover, the ERK1/2 inhibition also blocked p38 activation, indicating that ERK1/2 acts at the upstream of p38 and BMSC-exosomes regulate VEGF expression through ERK1/2-p38 MAPK pathways, resulting in enhanced tumor angiogenesis which in turn promotes the tumor growth* in vivo* [17].

### 5.3. MSCs-Derived Exosomes and Wound Healing

Angiogenesis is known to play a key role in the cutaneous wound healing process and is required for granulation tissue formation. During wound healing, angiogenic capillary sprouts invade the fibrin/fibronectin-rich wound clot and within a few days organize into a microvascular network throughout the granulation tissue [79]. Since previous works reported that MSCs-conditioned media can enhance wound healing, many studies have been performed evaluating the role of EVs in this process [80, 81]. However, the mechanisms behind the tissue repair are not completely understood.

In a recently reported study by Shabbir and colleagues [14], BMSCs-derived exosomes could enhance the growth and migration of normal and chronic wound fibroblasts and induce angiogenesis* in vitro*. The authors demonstrate that BMSCs-exosomes contained transcriptionally active STAT3, which could be partially involved in the transcription of genes related to the angiogenic process such as VEGF, hepatocyte growth factor (HGF), and IL-6. STAT3 have an important role in wound healing, including roles in migration, proliferation, angiogenesis, and growth factor production [82]. Furthermore, BMSC-exosomes were able to activate AKT, ERK 1/2, and STAT3, all signaling pathways involved in the regulation of angiogenesis [14].

MSCs derived from induced pluripotent stem cell (iPSC-MSCs) exosomes also have shown therapeutic effects in cutaneous wound healing through a significant enhanced collagen synthesis and the genesis of newly formed vessels and mature vessels in wound sites. iPSC-MSCs-exosomes were capable of promoting the proliferation and migration of human fibroblasts and HUVECs and enhanced the fibroblasts collagen and elastin secretion [83].

## 6. Conclusion

Exosomes through the delivery of molecules as proteins, mRNAs, and microRNAs mediate the angiogenic process. This property is highly influenced by their cell of origin, microenvironment, and the target cells. Therefore, their angiogenic activity can be modulated by screening the appropriate donor cells or microenvironment changes.

To date, progress in exosomes studies highlights the power of combining cell based therapies and their products with the engineering option of enhancing their content. With hundreds of clinical trials currently exploring the utility of stem cells, there is an emerging need to strengthen their potential in patients. Exosomes-based therapy could offer an acellular solution for bypassing many of the hurdles the cell strategy is locking: safety, potency, efficacy, and scalability of stem cell products. Finally, enhancing the exosomes with proangiogenic factors following different approaches offers new options for stem cell therapies to overcome the many translational barriers that the field is facing.

## Figures and Tables

**Table 1:** Characteristics of the different types of vesicles derived from different biological fluids and conditioned medium.

Vesicles	Characteristic				
	Origin	Size	Sucrose gradient	Markers	Contents
Exosomes	Endolysosomal pathway; luminal budding into multivesicular bodies (MVB) and release by fusion of MVB with cell membrane	40–120 nm	1.13–1.19 g/mL	Annexins, tetraspanins, heat-shock proteins, TSG101, flotillin, and MFGE8. They expose low amounts of phosphatidylserine and include cell-type-specific proteins	mRNA, miRNA, and noncoding RNAs; cytoplasmic and membrane proteins

Microvesicles	Cell surface; outward budding of cell membrane	50–1000 nm	1.04–1.07 g/mL	Integrins, selectins, and CD40 ligand	mRNA, miRNA, and noncoding RNAs; cytoplasmic and membrane proteins

Apoptotic bodies	Cell surface; outward blebbing of apoptotic cell membrane	1–5 *μ*m	1.16–1.28 g/mL	High amounts of phosphatidylserine	Nuclear fractions and cell organelles

**Table 2:** Summary of MSCs-derived exosomes in different studies.

Pathology	Model	Origin	Administration	Therapeutic effect	Reference
Cancer	BALB/c mice Syngeneic breast cancer model	Murine BMSCs-exosomes	2 × 10^5^ 4T1 cells mixed with 100 or 200 *μ*g exosomes	↓ expression of VEGF in tumor breast cells ↓ angiogenesis *in vitro* and *in vivo* ↓ tumor growth *in vivo*	[15]

Cancer	BALB/c nu/nu mice Xenogeneic gastric cancer model	BMSCs-exosomes	1 × 10^6^ SGC-7901 cells mixed with 200 *μ*g exosomes	↑ expression of VEGF in tumor breast cells ↑ angiogenesis *in vitro* and *in vivo* ↑ tumor growth *in vivo*	[17]

Wound healing	Cutaneous wound model in streptozotocin-induced diabetic rat	LPS-preconditioned UC-MSCs-exosomes	60 exosomes, injected into the wound edge	↓ inflammatory cell infiltration ↑ angiogenesis ↑ cutaneous wound healing	[46]

Stroke	Wistar rats Middle cerebral artery occlusion	Rat BMSCs-exosomes	100 *μ*g exosomes, intravenous	↑ neurite remodeling ↑ neurogenesis ↑ angiogenesis	[47]

Brain injury	Wistar rats Traumatic brain injury	Rat MSCs-exosomes	100 *μ*g exosomes, intravenous	↑ angiogenesis ↑ neurogenesis ↓ inflammation	[48]

CVD	Wistar rats Acute myocardial infarction	Human BM-MSCs EVs	80 *μ*g EVs, intramyocardial injection	↑ angiogenesis ↑ migration ↓ infarct size ↑ cardiac function	[49]

CVD	Sprague-Dawley rats Myocardial infarction	Rat BM-MSCs-exosomes	80 *μ*g exosomes, intramyocardial injection	↑ angiogenesis ↓ inflammation ↓ infarct size ↑ cardiac function	[50]

CVD	Sprague-Dawley rats Acute myocardial infarction	Human UC-MSCs-exosomes	400 *μ*g exosomes, intravenous	↑ angiogenesis ↑ migration ↓ apoptosis ↓ cardiac fibrosis ↓ infarct size ↑ cardiac function	[51]

CVD	Sprague-Dawley rats Myocardial infarction	Rat BM-MSCs overexpressed CXCR4 exosomes	Not specified	↑ angiogenesis ↓ infarct size ↑ cardiac function	[18]
